# An Innovative and Comprehensive Evaluation Strategy of an All-Ages and Whole of Community Nutrition Program

**DOI:** 10.1093/geroni/igaf122.1275

**Published:** 2025-12-31

**Authors:** Dalila Lanza, Madison LeCroy, Luisa Cardenas, R Gabriela Barajas-Gonzalez, Nelson Lin, Stella Yi, Celine Chan, Lan Doan

**Affiliations:** New York University Langone Health, New York, New York, United States; New York University Langone Health, New York, New York, United States; New York Academy of Medicine, New York, New York, United States; New York University Langone Health, New York, New York, United States; Joint Medical Program UC Berkeley Public Health, Berkeley, California, United States; New York University, New York, New York, United States; New York University Langone Health, New York, New York, United States; NYU Langone Health, New York, New York, United States

## Abstract

Harvest Share is a partnership between 11 multi-sector partners including NYU Langone Health, four farming/gardening organizations, three community-based organizations, two elementary schools, and a food pantry. The goal is to implement a whole-of-community intervention suitable for all ages to improve diet and social/built environments for English-, Chinese-, Spanish-, and Bangla-speaking community members living in Sunset Park and surrounding neighborhoods in Brooklyn, NY. This is achieved through a community-centered, equity-driven multi-level strategy centered on five main pillars: food access, nutrition education, experiential learning, food policy, and economic security. Much of the extant nutrition research is evaluated quantitatively and with clinical outcomes, which are limited in its ability to capture nuanced and social outcomes, especially over shorter time periods and for outcomes at the interpersonal or community levels. Furthermore, for community-engaged research or community-based participatory research programs, an important goal is to build equitable partnerships to address health outcomes and reduce disparities, which requires a multi-faceted evaluation strategy beyond individual-level outcomes examining participants. We describe Harvest Share’s innovative and comprehensive evaluation strategy to understand intended and unintended impacts the program had on community partners and program participants of all ages. The evaluation strategy included five mixed methods at multiple levels: 1) surveys and skin carotenoid assessment; 2) semi-structured interviews stratified by language and income level; 3) ripple effects mapping — a participatory method to collect untold stories and behind-the-scene activities that can ripple out from a specific program; 4) partnership evaluation and social networks analysis; and 5) process evaluation.

